# Open knee dislocation, triple intra-articular fractures and patellar tendon rupture: case report of a knee disaster treated with aggressive irrigation/debridement, early anatomic reduction and internal fixation

**DOI:** 10.1186/s12891-022-05268-y

**Published:** 2022-05-09

**Authors:** Farzad Vosoughi, Fardis Vosoughi, Seyed Hadi Kalantar

**Affiliations:** 1 Department of Orthopedic and Trauma Surgery, Joint Reconstruction Research Center, Imam Khomeini Hospital Complex, Tehran University of Medical Sciences, Tehran, Iran; 2 Department of Orthopedic and Trauma Surgery, Center of Orthopedic Trans-Disciplinary Applied Research (COTAR), Tehran University of Medical Sciences, Shariati hospital, Tehran, Iran; 3 Orthopedic Surgery Department, Joint Reconstruction Research Center, Imam Khomeini Hospital Complex, End of Keshavarz Blvd, 1419733141 Tehran, Iran

**Keywords:** Knee fracture dislocation, Trauma, Open fracture, External fixator

## Abstract

**Background:**

Open knee fracture-dislocation is a rare orthopedic injury. However, the importance of its correct management could not be overstated. To the best of our knowledge, this is the fifth study reporting a case with simultaneous Hoffa fracture and knee dislocation and the 1^st^ study describing a patient with open plateau fracture-dislocation accompanied with Hoffa fracture, patella fracture, and patellar tendon tear. In addition, this report is noticeable as our case had no gross ligament injury unlike frequent association of knee dislocation with knee collateral ligament damage.

**Case presentation:**

In this study, we describe a 34-year-old motorcyclist referred to our center following a motor car accident. Further work-up revealed an open irreducible posterolateral knee dislocation, type 5 Hohl and Moore plateau fracture, lateral femoral condyle Hoffa’s fracture, patellar fracture, and patellar tendon tear of his right knee. During an open reduction, it turned out that an entrapped lateral meniscus prevented the joint to be reduced by closed means. After applying a temporary external fixator, the patient was finally managed with open reduction and internal fixation.

**Conclusion:**

Irreducible knee dislocation needs further work up to rule out any interposed soft tissue into the joint. Aggressive irrigation/ debridement, early anatomic reduction, and internal fixation may help reduce open fracture complications including infection, non-union, and stiffness.

## Background

Knee dislocation is an uncommon orthopedic emergency accounting for less than 0.02% of orthopedic trauma [1] and 0.5% of joint dislocation [2]. Nearly 20% of knee dislocations are open and men are affected 4 times more often than women [3]. The dislocation may occur in anterior, posterior, lateral, medial or rotatory direction in 40%, 33%, 18%, 4% and 5% of cases respectively [4]. While anterior dislocation may injure the popliteal artery and threaten the limb vasculature, the posterior dislocation may be associated with entrapment of medial knee capsule, vastus medialis, gastrocnemius muscle or the meniscus into the joint causing any attempt at close reduction to fail [1]. Failure to address the soft tissue entrapment immediately may result in soft tissue necrosis, compartment syndrome and persistent disability [1]. In the following, we aim to present a patient with posterolateral irreducible open knee fracture dislocation accompanied with patellar tendon tear, plateau fracture and Hoffa fracture of the ipsilateral limb and delineate our management strategy along with clinical and radiographic outcomes. To the best of our knowledge, no case with similar combination of injuries has been reported in the literature. This report is in accordance with the CARE guideline and a written informed consent was obtained from the patient.

## Case presentation

**Fig. 1:**
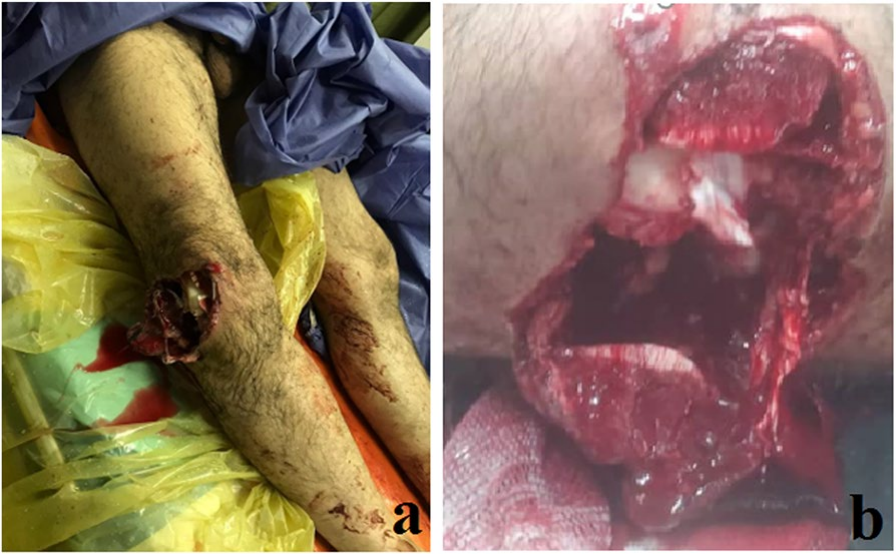
The initial presentation of our patient with an open knee fracture dislocation. He had a knee laceration on the anterolateral side and the distal femur was exposed

A 34-year-old motorcyclist was referred to our center after a high energy motor car accident. In the primary survey, our patient had normal airway and breathing with a blood pressure 100/60 and pulse rate 110/min. In the triage, two large bore 14-gauge IV lines were placed and he was treated with 1-L normal saline serum. His GCS was 15. Upon admission, he was conscious complaining of pain in his right knee. He had a visible laceration and flexion deformity of his right knee. He had no pain or complaint in any other parts of his body.He could not move his right knee. He had a 10 cm laceration on the anterolateral side of his right knee with irregular borders and a part of his distal femur was exposed through the wound (Fig. 1). Neurovascular assessment was normal. Ankle brachial index was obtained from the affected limb and its result was within normal limit (> 0.9). After 30 min from the patient’s arrival, he had blood pressure of 110/75 and pulse rate of 95/min and we continued with secondary survey.

**Fig. 2:**
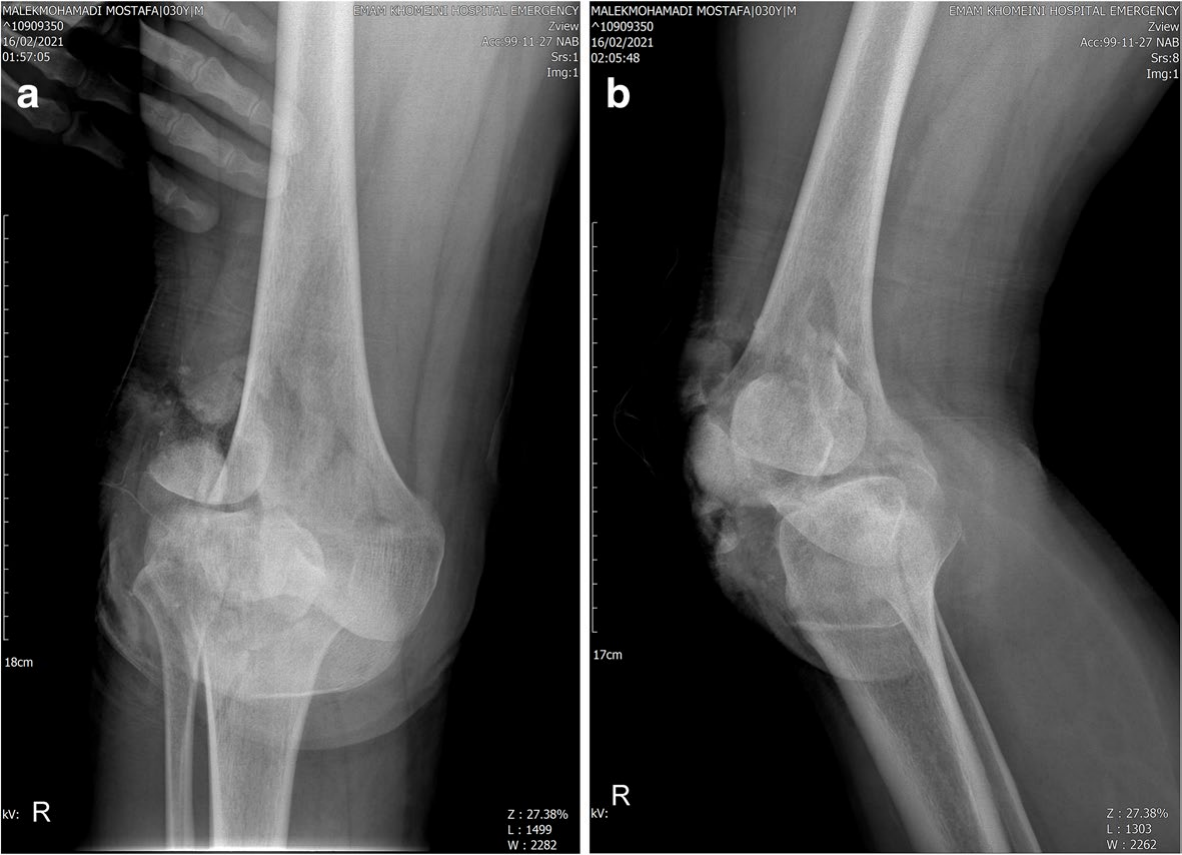
Plain knee X ray demonstrates a posterolateral fracture dislocation

**Fig. 3:**
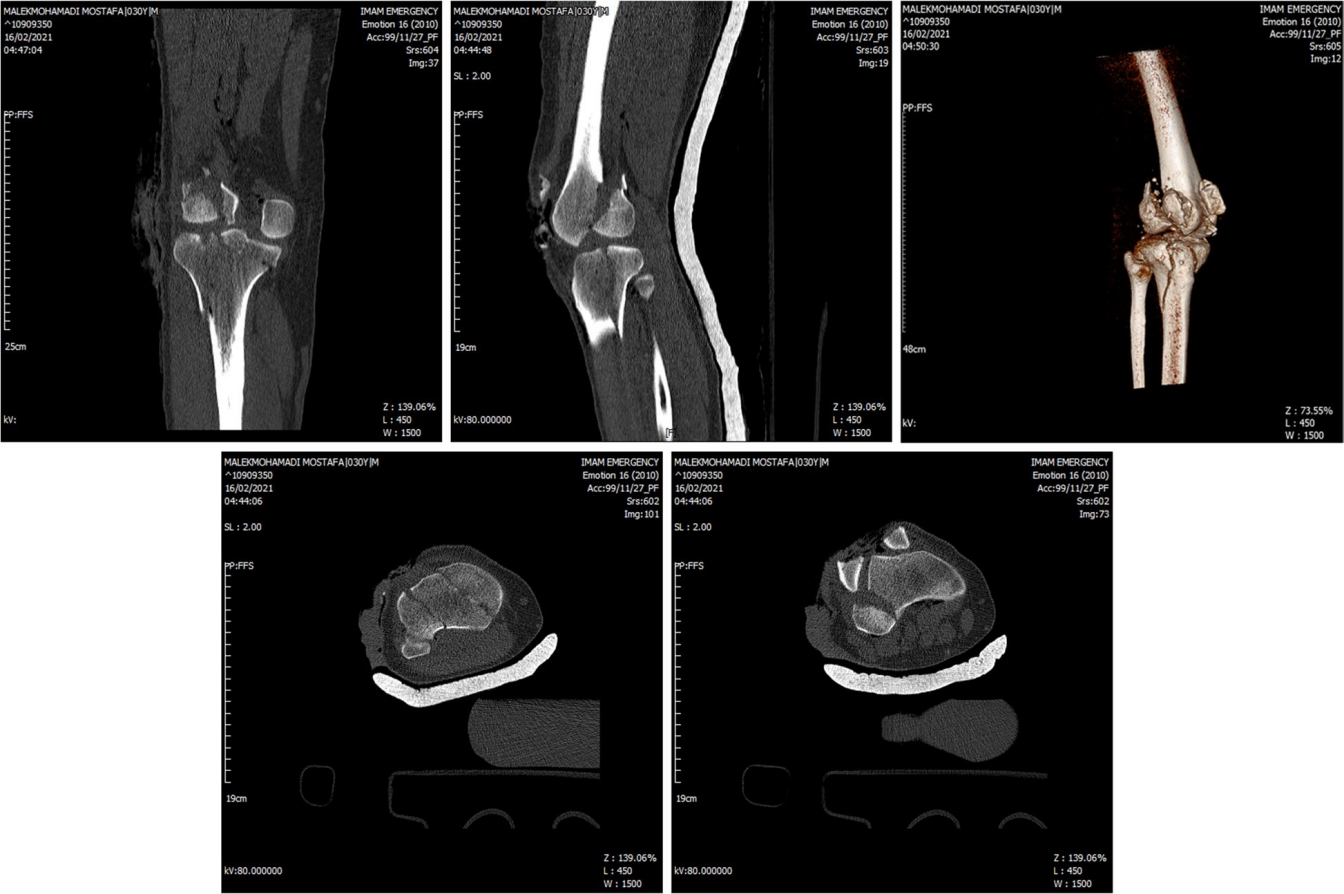
Computed Tomography revealed a posterolateral knee dislocation accompanied with lateral distal femoral Hoffa fracture, vertical patella fracture and type 5 Hohl and Moore fracture dislocation of the proximal tibia

**Fig. 4:**
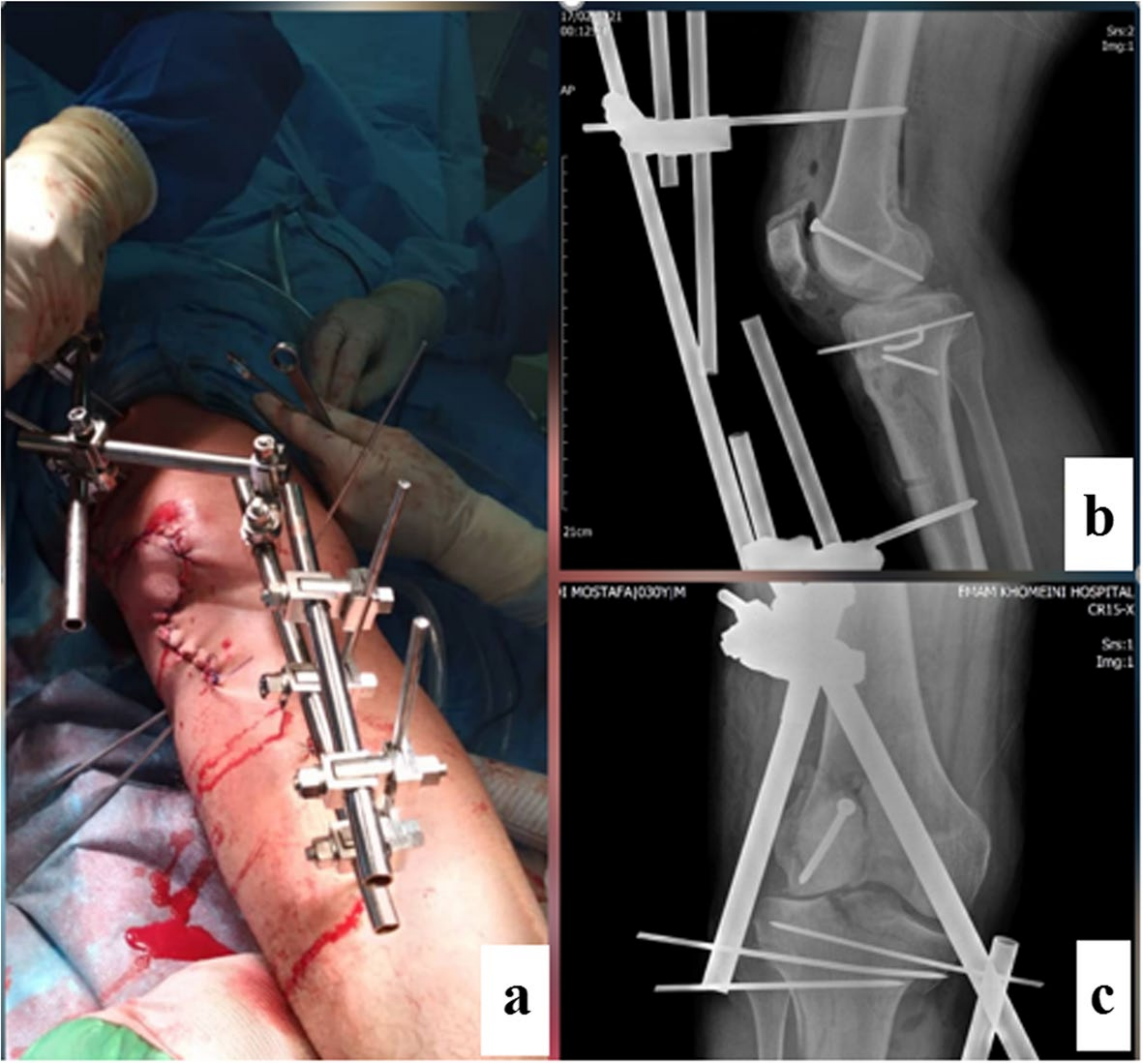
The patient’s wound after irrigation/ debridement. The Hoffa fracture was fixed with cancellous screw, the knee was reduced and temporary fixed with external fixator

In the secondary survey, gross foreign body and contaminations were cleaned from his wound, the laceration was irrigated with low pressure sterile normal saline and temporarily splinted. As the patient did not remember the timing of his tetanus prophylaxis, he was managed with a stat dose of tetanus vaccine, Tetabulin and prophylactic dosage of Cefazolin (2 mg, TDS) and Gentamicin (80 mg,BID). Given that his neurovascular exam was normal, we decided to perform his right knee imaging before any reduction attempt. X-rays revealed a posterolateral fracture dislocation of the knee (Fig. 2). CT images revealed a vertically oriented patella fracture (OTA classification [5], 34 B), lateral femoral condyle Hoffa’s fracture (OTA classification, 33B3.2) and a four part tibial plateau fracture dislocation according to the Hohl and Moore’s classification(OTA classification: 40A, KD 5) (Fig. 3). He was scheduled for emergent surgery 6 h after patient’s arrival to the hospital. His right knee was irrigated with 9-L normal saline and the remaining foreign bodies, dirt and necrotic tissue were debrided from the wound. The Hoffa’s and Hohl-Moore’s fractures were temporarily fixed with a 4 mm partially threaded cancellous screw and K wires respectively. The knee joint was reduced. However, as the reduction was not stable, the knee was stabilized temporary with bridging external fixator (Fig. 4). The wound was approximated and the patient was planned for a second look surgery 48 h later.

**Fig. 5:**
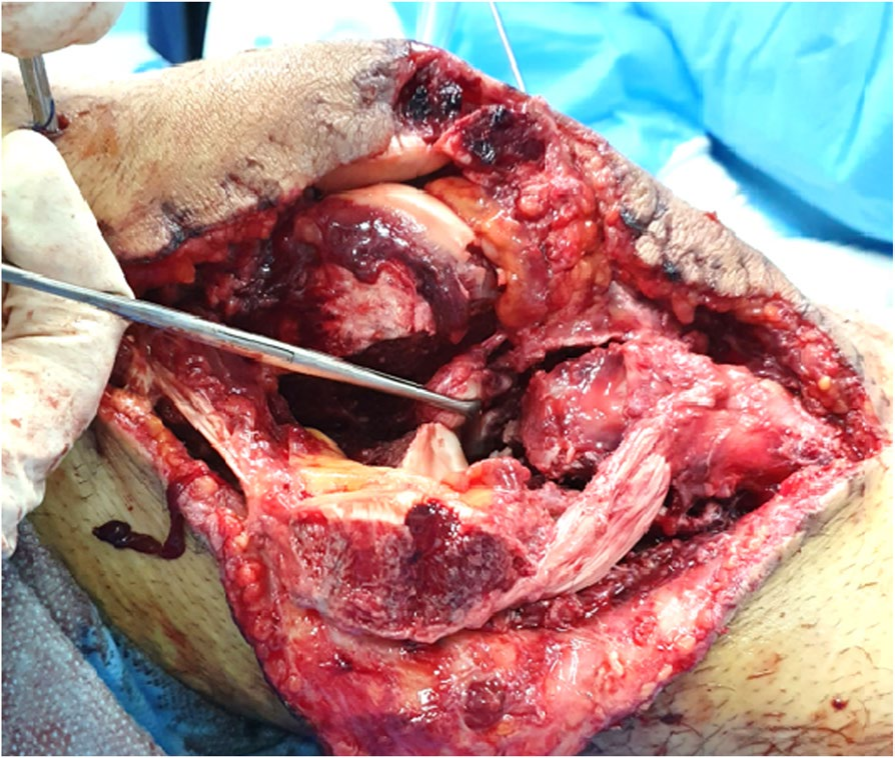
In the second look surgery, it was revealed that the lateral meniscus was interposed between proximal tibial fragments

In the 2^nd^ look surgery, the patient was positioned supine with a bump under the contralateral hip. He was fixed to operating table by thoracic and pelvic holders and tapes. After removing the sutures we carefully examined the wound. After making sure that there was no debris, no sign of early infection or remaining foreign body, crushed muscle or infected fascia, it was decided to convert external fixator to open reduction and internal fixation. During wound exploration, it was revealed that lateral meniscus was partially detached from its surrounding capsule and interposed between the proximal tibial fragments (Fig. 5). After reducing the lateral meniscus to its normal position, interestingly, the knee could be reduced into a stable anatomic fashion. Then we tilted the table to the right side so that the posteromedial side of the affected leg was exposed as if the patient was positioned in prone position. Then we managed the medial plateau fracture through posteromedial approach. The medial plateau was fixed with two reconstruction plates.

**Fig. 6:**
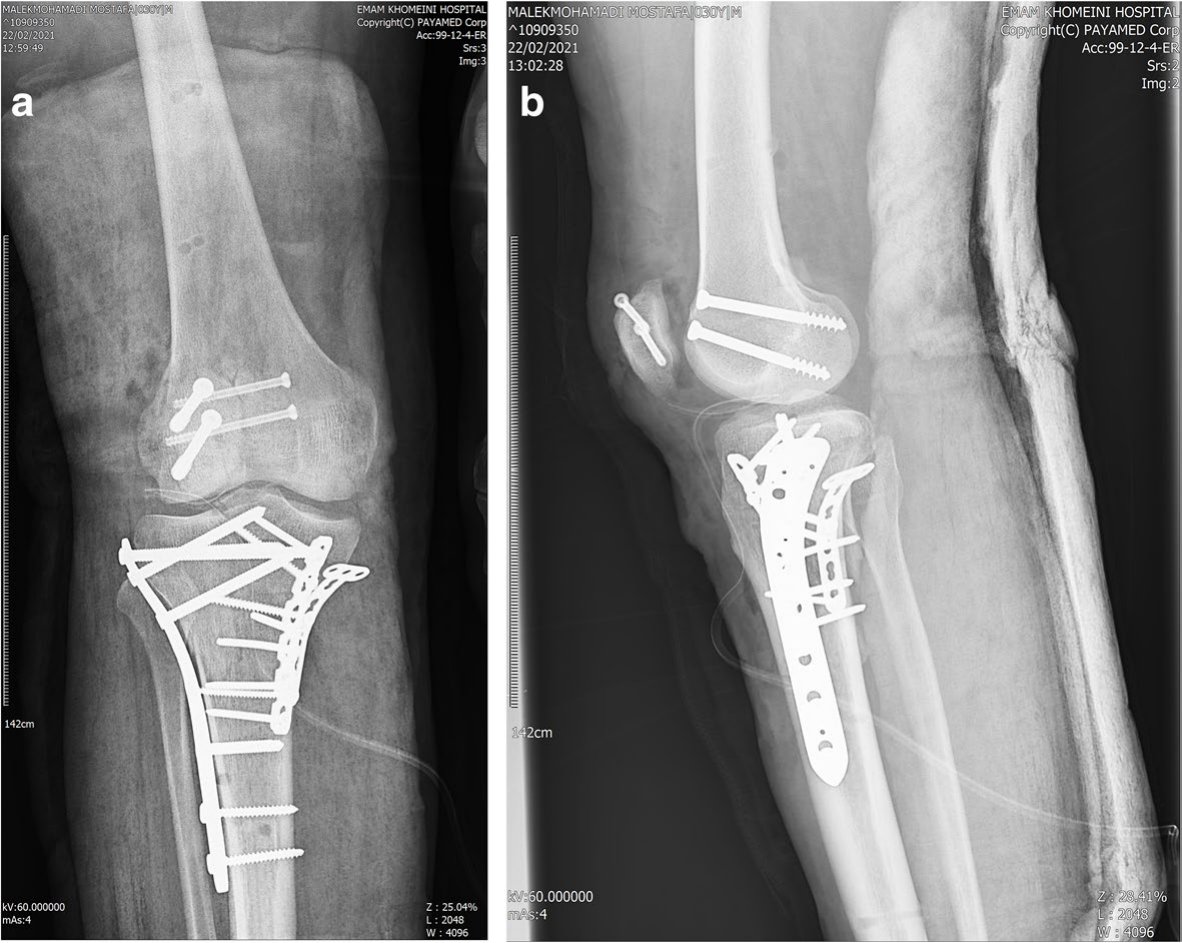
The final postoperative plain knee X ray of our patient

In the next step, the post was removed and his knee wound on the anterolateral side was exposed and extended. The lateral condyle Hoffa’s fracture was fixed with two 6.5 mm cannulated cancellous screws anteroposteriorly. Then, the lateral plateau was stabilized with a 4.5 locking compression plate (LCP). The lateral meniscus was repaired to its meniscocapsular junction with PDS 2 suture. The posteromedial approach was repaired and the anterolateral wound was approximated carefully not to cause more soft tissue and skin insults. As the 2^nd^ surgery was performed with tourniquet (tourniquet time 120 min), we worried that managing patellar fracture and patellar tendon injury in the same session, would risk the limb perfusion and cause more soft tissue damage or compartment syndrome. Therefore, we decided to address the patellar fracture in a different session 1 day after the second surgery. During the 3^rd^ operation, the vertical patellar fracture was fixed with two horizontal 4 mm cancellous screws. It was observed that the patellar tendon was also torn vertically. The torn patellar ligament was repaired with non-absorbable sutures and the wound was closed (Fig. 6). Following fixation, the patient’s anterior drawer test, posterior drawer test, valgus/ varus stress tests were normal and the knee was stable.

**Fig. 7:**
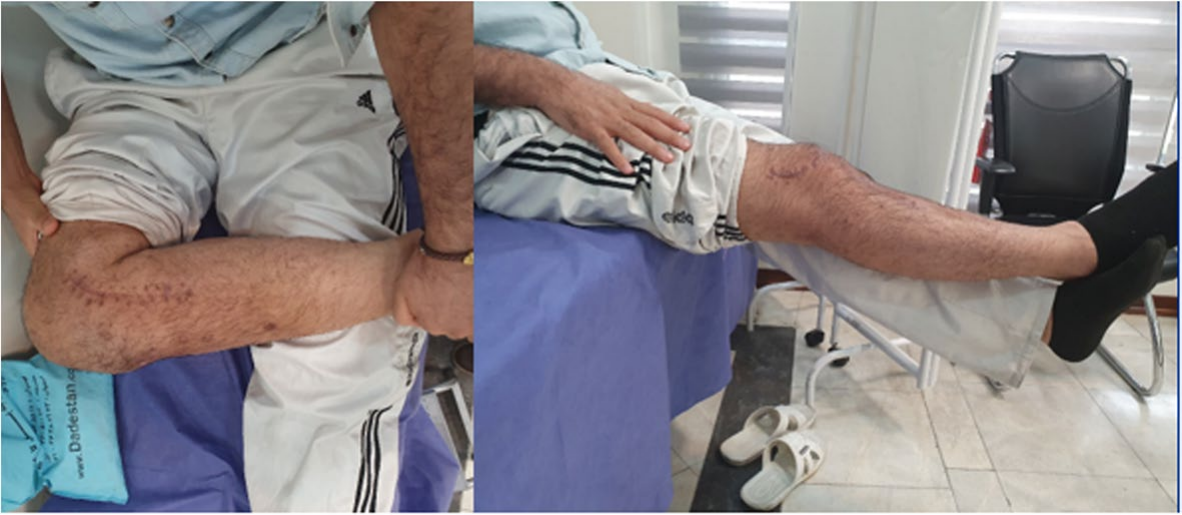
Our patient regained his knee range of motion from nearly complete extension to 120 degree flexion

**Fig. 8:**
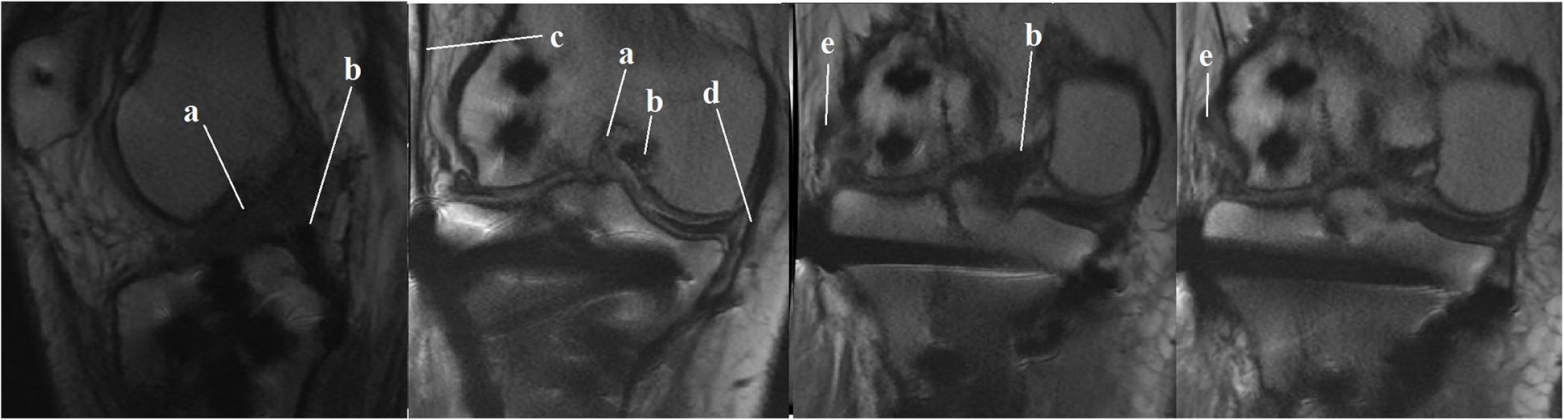
MRI at the latest follow up: a, anterior cruciate ligament; b,posterior cruciate ligament; c, tensor fascia lata; d, medial collateral ligament; e, fibular collateral ligament

Postoperatively, the patient was immobilized in full extension with a hinge knee brace for 2 weeks (except during rehabilitative sessions). After 3 days, active knee flexion and passive knee extension was started. At 2 weeks postoperatively, the patient was able to flex the knee 90 degrees. By physiotherapy, his knee flexion further improved to 120 degrees at 6-week post-surgery. At that time, active knee extension and partial weight bearing was started and the brace was unlocked. After 10 weeks, the brace was discontinued. Three months after the operation, our patient had a remaining 10-degree extension lag which was corrected till the 4^th^ month. At that time, as the fracture union was evident on the patient’s plain X-ray, full weight-bearing was started. At our last follow-up 15 months after the operation, he could flex the knee to 120 degrees and extend his joint completely. (Fig. 7). He, fortunately, was able to bear weight without any pain or limping and was satisfied with the treatment. At 15 month postoperatively, except for a partial ACL injury, the patinet’s MRI did not show any other associated soft tissue lesion (Fig. 8).

## Discussion

Open knee dislocation along with patellar tendon tear is relatively uncommon scenario and many orthopedic surgeons may not have encountered it throughout their career. Simultaneous occurrence of Hoffa’s fracture and knee dislocation is also quite rare [6, 7] and we found only 4 papers reporting such injuries [6, 8–10]. To the best of our knowledge, this study reports the first case of a patient with simultaneous Hoffa fracture, knee dislocation, patellar tendon tear and Plateau fracture.

One of the interesting novel points in our patient was the interposition of lateral meniscus between tibial bone fragments. After reducing the meniscus, the joint was stabilized and reduced. This emphasizes the need for careful joint exploration in unstable or irreducible knee dislocation.

Regarding our patient, we repaired the lateral meniscus to its meniscocapsular junction using PDS suture. We found another study describing a similar technique for managing meniscus injury encountered during Plateau fracture fixation [11]. However, more studies may be needed to determine the best management option.

Taking our patient’s radiologic imaging (Fig. 2) into account, he had a type 5 Hohl and Moore knee fracture dislocation. Interestingly, after fracture fixation, the joint was stable in physical examination (negative anterior/ posterior drawer test, negative varus/ valgus stress test) and no gross ligament injury was detected. There is a case report in the literature describing a posteromedial knee fracture dislocation scenario with no knee ligament injury [12]. We think our patient’s associated articular fractures may protected knee ligaments from severe damage.

We also had challenges in managing our patinet’s open knee fracture dislocation in terms of the chosing irrigation (low pressure vs high pressure, in the emergency vs in the operating room) and fixation technique (external fixator vs internal fixation), the time for conversion from temporary fixation into final fixation (< 1 week vs > 1 week), the sequence of fixation, and last but not the least, in the time of staring range of motion.

The management of an open fracture-dislocation has changed significantly in recent years and its treatment is still controversial [13]. In the Fluid Lavage of Open Wound (FLOW) clinical trial, the reoperation rate following high pressure (> 20 psi), low pressure (5–10 psi) and very low pressure (1–2 psi) irrigation for open fracture were evaluated. They did not find any significant difference between the options with regard to the rate of reoperation. They concluded that low pressure irrigation can be a low cost and reasonable option in the management of open fracture [14]. Irrigating the wound with high pressure lavage may displace the wound contamination through deeper structures (e.g. the joint space) [15]. Therefore, we applied low pressure pulsatile lavage for cleaning the laceration. We also tried to eliminate any contamination and foreign body before attempting reduction in order to prevent contaminating the deep soft tissue and joint space during reduction.

Regarding fixation technique, in the first surgery, as the joint reduction was not stable (the interposition of the lateral meniscus was not detected in the first surgery) and the joint was open and contaminated, we used external fixation to stabilize the joint and prevent possible neurovascular compromise. However, the time of conversion (after < 7 vs > 7 days) from external fixation to definite internal fixation was a challenging clinical dilemma. Early conversion has a risk that contamination might not be eliminated completely leading to surgical site infection. Late conversion could cause joint stiffness, pin-tract infection, bed sore and deep vein thrombosis.

Some papers stressed the improved outcome of early conversion (within 4–7 days) over delayed conversion. Delayed option was associated with up to 50% chance of infection while the early switch to internal fixation had lower infection rate (nearly 5%) [16–18]. Also, delayed conversion may become more complicated by pin loosening and pin-tract infection. Nowotarsky et. al. studied the outcome of multiple trauma cases having femoral fracture. They used external fixator within 24 h and changed it to internal fixation within an average of 7 days. They reported an infection rate of 1.7% and unplanned reoperation of 11% [19]. The recent consensus did not suggest a definite time limit for conversion. However, in the absence of pin site infection, it recommended an early one stage conversion to definite internal fixation [15]. Regarding our patient, as the wound was judged to be clean in the second surgery and no sign of pin tract infection was noticed, we decided to switch to definite internal fixation early (within 48 h).

Even with recent advances in management, infection, non-union and poor functional outcome remain as serious complications of open fractures [20]. In a study of tibial Plateau fracture, Kugelman et. al. demonstrated that open fracture, multiple trauma, tibial spine fracture, applying knee spanning external fixator, and postoperative infection are the modifiable risk factors for postoperative knee stiffness [21]. Though our patient had many of the mentioned risk factors (applying knee spanning external fixator, open fracture), we believe that the aggressive management including fast transmission from external fixation to internal fixation and early initiation of range of motion might have a role in preventing further knee stiffness in our patient. However, further prospective studies may give a better perspective of the management of knee fracture dislocation.

There are few reports on open knee fracture dislocations. Therefore, we think that this report, its literature review and follow up could be beneficial in managing such complicated emergency scenarios. However, higher level of evidence with larger sample size could pave the way to better understanding of open knee fracture dislocation treatment.

## Conclusion

Irreducible knee dislocation needs further work up to rule out any interposed soft tissue into the joint. Such disastrous cases should not corner the surgeon toward prolonged immobilizations. Aggressive irrigation/ debridement, early anatomic reduction, and internal fixation may help reduce open fracture complications including infection, non-union, and stiffness.

## Data Availability

Data sharing is not applicable to this article as no datasets were generated or analyzed during the current study.

## Abbreviations

CARE: Case Report
ATLS: Advanced Trauma Life Support
CT: Computed Tomography
OTA: Orthopaedic Trauma Association; mm, millimeter
LCP: Locking Compression Plate
ACL: Anterior Cruciate Ligament
MRI: Magnetic Resonance Imaging
FLOW: Fluid Lavage of Open Wound
PDS: Polydioxanone suture
psi: Pounds per square inch

## References

[1] Malik SS, MacDonald PB (2020). The Irreducible Knee Dislocation. J Knee Surg.

[2] Durakbaşa MO, Ulkü K, Ermiş MN (2011). Irreducible open posterolateral knee dislocation due to medial meniscus interposition. Acta Orthop Traumatol Turc.

[3] Manske RC, Hosseinzadeh P, Giangarra CE (2008). Multiple ligament knee injury: complications. N Am J Sports Phys Ther.

[4] Green NE, Allen BL (1977). Vascular injuries associated with dislocation of the knee. J Bone Joint Surg Am.

[5] Meinberg EG, Agel J, Roberts CS, Karam MD, Kellam JF (2018). Fracture and dislocation classification compendium—2018. J Orthop Trauma.

[6] Huang G, Zhang M, Zhang Y, Wang X, Zhang M, Liu G (2021). Hoffa fracture combined with rotational dislocation of the knee joint: A novel case report. Med (Baltimore).

[7] Calderazzi F, Visigalli A, Scita G, Spirito A, Ferrari U, Ceccarelli F, Pogliacomi F (2021). Open fracture-dislocation of the knee associated with nonunion of the medial femoral condyle and chronic tendon patellar rupture. Acta Biomed.

[8] Schenck RC, McGanity PL, Heckman JD (1997). Femoral-sided fracture-dislocation of the knee. J Orthop Trauma.

[9] Shetty GM, Wang JH, Kim SK, Park JH, Park JW, Kim JG, Ahn JH (2008). Incarcerated patellar tendon in Hoffa fracture: an unusual cause of irreducible knee dislocation. Knee Surg Sports Traumatol Arthrosc.

[10] Shah Faaiz Ali, Ali Main Amjad, Qureshi Abdur Rehman, Naeemullah, Khan Umar Zia, Sarwar. KM: A Rare Variant of Acute open Fracture Dislocation of Knee Joint- A Case Report. JIMDC 2018, 7(1):78–82.

[11] Durakbasa MO, Kose O, Ermis MN, Demirtas A, Gunday S, Islam C (2013). Measurement of lateral plateau depression and lateral plateau widening in a Schatzker type II fracture can predict a lateral meniscal injury. Knee Surg Sports Traumatol Arthrosc.

[12] Green RN, Pullagura MK, Holland JP (2014). Irreducible fracture-dislocation of the knee. Acta Orthop Traumatol Turc.

[13] Gümbel D, Matthes G, Napp M, Lange J, Hinz P, Spitzmüller R, Ekkernkamp A (2016). Current management of open fractures: results from an online survey. Arch Orthop Trauma Surg.

[14] Bhandari M, Jeray KJ, Petrisor BA, Devereaux PJ, Heels-Ansdell D, Schemitsch EH, Anglen J, Della Rocca GJ, Jones C, Kreder H (2015). A Trial of Wound Irrigation in the Initial Management of Open Fracture Wounds. N Engl J Med.

[15] Baril WC: ICM PHILLY: Trauma. 2018.

[16] Blachut PA, Meek R (1990). O'brien P: External fixation and delayed intramedullary nailing of open fractures of the tibial shaft. Sequential Protoc JBJS.

[17] McGraw J, Lim E (1988). Treatment of open tibial-shaft fractures. External fixation and secondary intramedullary nailing. J Bone Joint Surg Am Vol.

[18] Fischer MD, Gustilo RB, Varecka T (1991). The timing of flap coverage, bone-grafting, and intramedullary nailing in patients who have a fracture of the tibial shaft with extensive soft-tissue injury. J Bone Joint Surg Am.

[19] Nowotarski PJ, Turen CH, Brumback RJ, Scarboro JM (2000). Conversion of external fixation to intramedullary nailing for fractures of the shaft of the femur in multiply injured patients. JBJS.

[20] Elniel AR, Giannoudis PV (2018). Open fractures of the lower extremity: Current management and clinical outcomes. EFORT Open Rev.

[21] Kugelman DN, Qatu AM, Strauss EJ, Konda SR, Egol KA (2018). Knee Stiffness After Tibial Plateau Fractures: Predictors and Outcomes (OTA-41). J Orthop Trauma.

